# Cannabinoids in Chronic Pain Management: A Review of the History, Efficacy, Applications, and Risks

**DOI:** 10.3390/biomedicines13030530

**Published:** 2025-02-20

**Authors:** Brooks W. Johnson, Natalie H. Strand, John C. Raynak, Christian Jara, Kisanet Habtegiorgis, Brennan A. Hand, Sang Hong, Jillian A. Maloney

**Affiliations:** 1 Department of Anesthesiology and Perioperative Medicine, Mayo Clinic, Phoenix, AZ 85054, USAraynak.john@mayo.edu (J.C.R.); maloney.jillian@mayo.edu (J.A.M.); 2 Mayo Clinic Alix School of Medicine, Phoenix, AZ 85054, USA; 3 Creighton University School of Medicine, Phoenix, AZ 85012, USA; sanghong@creighton.edu

**Keywords:** cannabinoids, chronic pain, tetrahydrocannabinol, marijuana, headache, neuropathy, fibromyalgia

## Abstract

**Background/Objectives**: Chronic pain remains a pervasive and challenging public health issue, often resistant to conventional treatments such as opioids, which carry substantial risks of dependency and adverse effects. Cannabinoids, bioactive compounds derived from the Cannabis sativa plant and their synthetic analogs, have emerged as a potential alternative for pain management, leveraging their interaction with the endocannabinoid system to modulate pain and inflammation. **Methods**: The current, evolving literature regarding the history, efficacy, applications, and safety of cannabinoids in the treatment of chronic pain was reviewed and summarized to provide the most current review of cannabinoids. **Results**: Evidence suggests that cannabinoids provide moderate efficacy in managing neuropathic pain, fibromyalgia, cancer-related pain, and multiple sclerosis-related spasticity. Patient-reported outcomes further indicate widespread perceptions of cannabinoids as a safer alternative to opioids, with potential opioid-sparing effects. However, the quality of existing evidence is limited by small sample sizes and methodological inconsistencies. Regulatory barriers, including the classification of cannabis as a Schedule I substance in the United States, continue to hinder robust research and clinical integration. Moreover, the risks associated with cannabinoids, such as psychiatric effects, addiction potential, and drug interactions, necessitate cautious application. **Conclusions**: Cannabinoids represent a promising, albeit complex, alternative for chronic pain management, particularly given the limitations and risks of traditional therapies such as opioids. However, significant deficiencies remain in the research. While smaller trials and systematic reviews indicate therapeutic potential, the quality of evidence is often low due to limited sample sizes, short study durations, and methodological inconsistencies. Large-scale, randomized controlled trials with long-term follow-up are urgently needed to confirm efficacy and safety across diverse patient populations and pain etiologies.

## 1. Introduction

The clinical practice of pain management encompasses a variety of strategies, including pharmacological, interventional, and psychological approaches. Pain itself is a multifaceted symptom, often categorized into acute and chronic types, where chronic pain persists beyond standard tissue healing time and poses significant challenges to conventional treatment methods. Modern approaches to pain management include a combination of over-the-counter analgesics, prescription medications, physical therapy, lifestyle modifications, and interventional therapies. However, these strategies often yield mixed results, particularly for chronic pain conditions, as some patients develop tolerance or experience side effects [1].

Pain and its effects on those afflicted represent a significant public health challenge. According to the World Health Organization (WHO), pain is a leading cause of disability, impacting social and occupational function [2]. Chronic pain conditions, such as arthritis, musculoskeletal pain, headaches, fibromyalgia, and neuropathic pain, affect nearly one in five individuals globally, often leading to a diminished quality of life, economic hardship, and mental health issues, including depression and anxiety [2]. Despite advancements in medical science, pain management remains inadequately addressed in many healthcare systems, with disparities in access to adequate pain relief across countries and socioeconomic groups [3].

## 2. Opioids and the Epidemic

For decades, opioids have been the cornerstone of moderate-to-severe pain management. While opioids are effective in reducing pain, their use is fraught with the risk of tolerance, dependence, and potential for abuse. The overprescription of opioids has contributed to a global health crisis, particularly in the United States, where opioid misuse and overdose deaths have surged [4]. The opioid epidemic has directly led to substantial loss of life, economic costs, and worsening healthcare outcomes. Since its onset in the late 1990s, the misuse of prescription opioids has played a central role in escalating this epidemic. The opioid crisis has claimed over 70,000 lives annually in recent years, with prescription opioids accounting for a significant portion of these deaths. Between 2015 and 2020, fatalities related to prescription opioid overdoses quadrupled [5].

Economically, the epidemic has imposed an estimated staggering annual cost of over USD 1 trillion in the United States, including the costs from healthcare expenses, loss of productivity, criminal justice, social welfare interventions, reduced quality of life for opioid use disorder, and life lost due to fatal opioid overdose [6]. This estimate includes the costs associated with treating opioid use disorder (OUD) and addressing its far-reaching and long-lasting effects.

Despite increasing awareness, prescriptions for opioids have remained high in certain contexts, contributing to both initiation of misuse and prolonged dependency. The prolonged use of opioids in managing chronic pain can lead to dependency, increasing the risk of patients transitioning from prescribed use to illicit drug consumption when prescriptions are no longer accessible [7]. The management of pain with opioids is not without risk or significant side effects. Independent of the inherent risk of opioid use disorder and dependence, the chronic administration of opioids is associated with other adverse effects, including increased risk of falls and fractures, reduced immune response, hypogonadism and resultant sexual dysfunction, and, in multiple studies, increased risk of myocardial infarction [8].

While opioids certainly have a place in the treatment of acute and chronic pain, the need for novel, effective pain therapies is more pressing than ever.

Cannabinoids, the active compounds in the Cannabis sativa plant, have emerged as a potential therapeutic adjunct and alternative for pain management due to their analgesic and anti-inflammatory properties. These compounds interact with the body’s endocannabinoid system, modulating pain and inflammation at multiple physiological levels [9].

## 3. History of Cannabinoids for the Treatment of Pain

The use of cannabis for medicinal purposes can be traced back centuries in several regions of the world. Some of the earliest recorded uses date to 2900 BC in China, where the common name for hemp and cannabis appeared in compositions describing numbness or anesthesia [10]. Different utilizations of medicinal cannabis are present in ancient writings; one notable example is found in the Ebers Papyrus from Egypt, written around 1500 BC, which discusses the topical application of cannabis for inflammation [11]. Similarly, writings from the Roman Empire, particularly those of Pliny the Elder in The Naturalis Historia, document the antalgic and anti-inflammatory properties of cannabis in treating ailments such as arthritis and gout [12].

In the early 1800s, Dr. William O’Shaughnessy, a surgeon serving in India, published a medical pamphlet describing the narcotic and psychoactive effects of hemp in various forms. These effects were well known across Asia and Africa but unfamiliar to Western Europe. His writings highlighted the use of cannabis in large quantities to alleviate symptoms of hydrophobia and paroxysms in tetanus. Observed effects also included anticonvulsive and analgesic properties in the populations of India [10]. Following this publication, cannabis became widely available as an over-the-counter medication in Europe and the United States. Cannabis was used to treat various ailments, including headaches, anorexia, tetanus, rabies, dysentery, cholera, and opiate addiction, and served as a sleep aid. By 1890, Sir Russell Reynolds, who would later preside over the British Royal Society of Medicine, promoted the use of cannabis for migraines and neuralgias with patented marijuana tinctures readily available [13].

As public concerns about addiction to intoxicants such as opium grew, regulations and laws targeting such substances emerged. In 1906, the Pure Food and Drug Act mandated the regulation of labeling and dosages for medications containing cannabis, cocaine, or morphine [14]. State legislation soon followed, ultimately banning over-the-counter cannabis in all 50 U.S. states. At the federal level, the Marijuana Tax Act of 1937 imposed taxes on the production, distribution, and possession of cannabis, even for research purposes, effectively ending its use in the United States [15]. Cannabis was subsequently removed from the United States Pharmacopoeia in 1942, with legal penalties for possession increasing in 1951 and 1956 with the enactment of the Boggs and Narcotic Control Acts, respectively, and prohibition under federal law, stemming from the “war on drugs” occurring with the Controlled Substances Act of 1970 [16,17].

Despite these regulatory hurdles, the discovery of cannabinoid receptors in the 1990s marked a significant turning point in understanding the potential medical benefits of cannabis. During this period, oncologists and medical organizations [18], including the American Medical Association, advocated for the reclassification of cannabis and its legalization for prescription use. These efforts reignited debates about the therapeutic potential of cannabis and its place in modern medicine. Since then, research targeting the therapeutic use of cannabis, and its components, has flourished.

## 4. Regulatory Landscape

### 4.1. United States

The classification of cannabis as a Schedule I substance under the Controlled Substances Act presents significant regulatory barriers to its use in pain management, primarily due to the stringent restrictions associated with this designation. Schedule I status implies that cannabis has a high potential for abuse, no accepted medical use, and a lack of accepted safety under medical supervision [19]. This classification imposes substantial hurdles for researchers, who must navigate a complex and costly approval process to study cannabis, often requiring multiple levels of federal approval and oversight [20]. These regulatory challenges limit the quantity and quality of research that can be conducted, thereby hindering the development of evidence-based clinical applications for cannabinoids in pain management. Additionally, the Schedule I status restricts physicians’ ability to prescribe cannabis, limiting patient access to potentially beneficial treatments and forcing them to rely on less regulated and often less safe alternatives [21]. This regulatory environment creates a significant gap between the growing anecdotal evidence supporting the efficacy of cannabis in pain relief and the robust clinical data needed to inform medical practice and policy. Addressing these barriers is crucial for advancing the scientific understanding and clinical integration of cannabinoids in pain management.

On 21 May 2024, the U.S. Department of Justice issued a notice of proposed rulemaking to reclassify marijuana under the Controlled Substances Act (CSA), moving it from a Schedule I to Schedule III substance. This action aligns with the Department of Health and Human Services’ (HHS) determination that marijuana has a recognized medical use and a lower potential for abuse compared to substances listed in Schedules I and II [22]. A change in the scheduling of marijuana, and its psychoactive components, would certainly open the doors to further research.

Regulatory approaches to cannabinoid-based treatments vary significantly across different countries and regions, influencing both research progress and clinical implementation. While the United States has historically imposed strict federal restrictions, other regions such as Canada and Europe have adopted more flexible regulatory frameworks, facilitating broader clinical use and scientific exploration.

### 4.2. Canada

Canada has been a global leader in cannabis regulation, having fully legalized both medical and recreational cannabis through the Cannabis Act of 2018 [23]. Prior to this, Canada established a structured medical cannabis program in 2001, permitting physicians to authorize cannabis for patients under Health Canada’s oversight [24]. The country’s regulatory environment has allowed for more extensive clinical research on cannabinoids, with institutions receiving government funding for studies on their efficacy in treating chronic pain [25].

### 4.3. Europe

Europe presents a diverse regulatory landscape, with policies varying by country. Nations such as Germany, the Netherlands, and Portugal have established medical cannabis programs, allowing physicians to prescribe cannabinoids for chronic pain and other conditions [26]. In Germany, the 2017 Cannabis as Medicine Act enabled widespread insurance reimbursement for medical cannabis, significantly increasing patient access [27]. The Netherlands, through its Office of Medicinal Cannabis, has long supported regulated production and distribution, facilitating research efforts beginning with the first-of-its-kind national medicinal cannabis program starting in 2003 [28].

Conversely, some European nations maintain restrictive policies. France and Sweden, for example, have only recently begun controlled trials of medical cannabis, reflecting a more cautious regulatory stance [29,30].

### 4.4. Impact on Research

In contrast to the U.S., where federal restrictions have historically hindered large-scale research, Canada and parts of Europe have fostered more research-friendly environments.

By integrating these international perspectives, it is evident that regulatory frameworks play a crucial role in shaping the availability and scientific understanding of cannabinoid therapies for chronic pain. A comparative approach highlights both the challenges and opportunities that different regulatory models present for advancing cannabinoid-based medicine.

## 5. Cannabinoids and the Endocannabinoid System

Cannabinoids are a diverse group of bioactive compounds predominantly derived from the Cannabis sativa plant, known for their interaction with the endocannabinoid system in the human body. They include phytocannabinoids, those produced by plants of the Cannabis genus, include tetrahydrocannabinol (THC), cannabidiol (CBD), and dronabinol [31]. Synthetic and endogenous variants have also been characterized and studied, including anandamide and 2-arachidonoylglycerol (2-AG). Cannabinoids are characterized by a terpenophenolic chemical structure, typically comprising a bicyclic or tricyclic arrangement with a hydroxyl group and various side chains that determine their receptor affinity and pharmacological effects [32]. Beyond their therapeutic effects, cannabinoids remain a focal point of research due to their complex biosynthesis, involving precursor molecules like cannabigerolic acid (CBGA) that form under enzymatic catalysis within cannabis trichomes [33].

Cannabinoid receptors are essential components of the body’s endocannabinoid system, regulating various physiological functions such as pain perception and modulation. Additionally, the endocannabinoid system contributes to homeostasis and neuroplasticity, playing a role in neurogenesis and the refinement of neuronal connections [34]. Endocannabinoids are linked to improved emotion regulation, a decrease in physiological stress response, and increased reward pathway signaling [35]. The two most relevant cannabinoid receptors, the CB1 and CB2 receptors, are Gi/o subtypes of G proteins that are negatively coupled to adenylate cyclase and positively coupled to mitogen-activated protein kinase (MAPK) [36].

CB1 receptors are distributed throughout the central nervous system and peripheral tissues, with particularly high concentrations in the brain including the cerebellum, hippocampus, basal ganglia, amygdala, cerebral cortex, and spinal cord [37]. CB1 receptors regulate cannabinoid neurotransmitter effects within the central nervous system by inhibiting neuronal excitation and activity. The activation of CB1 receptors reduces the release of excitatory neurotransmitters, such as glutamate, while promoting the activation of pain-modulating circuits by inhibiting the release of inhibitory neurotransmitters such as gamma-aminobutyric acid (GABA) [38]. Additionally, evidence suggests that CB1 receptors inhibit the release of other neurotransmitters, including acetylcholine, noradrenaline, dopamine, 5-hydroxytryptamine (5-HT), D-aspartate, and cholecystokinin [38]. Through the regulation of neurotransmitter release, CB1 receptors play a significant role in pain modulation, influencing neuronal excitability and synaptic plasticity.

CB2 receptors have been classically thought to be primarily involved in the immune response and are found in the periphery, particularly in hematopoietic stem cells, macrophages, and other immune cells [39]. These receptors regulate neuroimmune interactions in response to pathogens, adjust the sensitivity of sensory neurons to painful stimuli, and respond to peripheral nerve injuries. The CB2 receptor exerts its anti-inflammatory effects by inhibiting pro-inflammatory cytokines, such as IL-1β and TNF-α, while simultaneously promoting the secretion of anti-inflammatory mediators. CB2 receptor activation [40,41], often triggered by endogenous cannabinoids like 2-AG, involves pathways such as the inhibition of adenylate cyclase, calcium channel deactivation, and stimulation of the MAPK/ERK signaling cascade. This receptor also mitigates neuroinflammation by modulating microglial activation, reducing blood–brain barrier permeability, and protecting against cytokine-induced neuronal damage [40]. These properties make the CB2 receptor a promising target for therapeutic interventions in chronic inflammatory diseases, neuropathic pain, and neurodegenerative disorders [42].

The interaction between cannabinoids and neurotransmitter systems plays a significant role in pain perception and modulation. Endocannabinoids, such as anandamide and 2-AG, are endogenous lipid-based retrograde neurotransmitters that act as synaptic messengers by interacting with cannabinoid receptors CB1 and CB2 during stress or tissue injury to regulate nociceptive signaling. Anandamide interacts with CB1 receptors to inhibit neurotransmitter release, which is key in processing nociceptive information. Similarly, 2-AG acts as an agonist for CB1 receptors in response to tissue injury, effectively modulating pain during episodes of acute stress [43].

**Figure 1:**
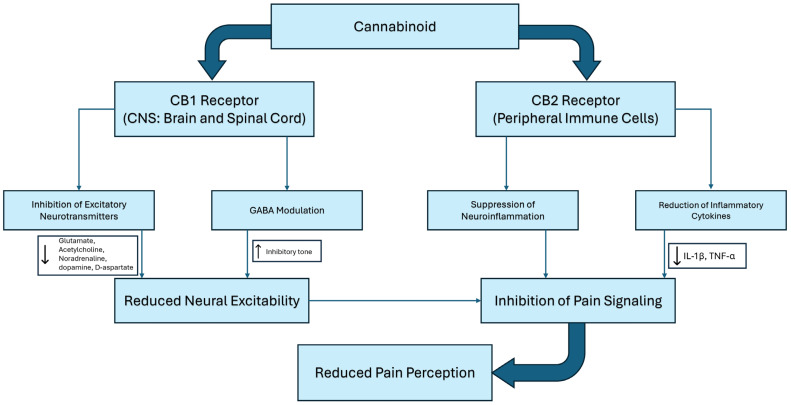
Proposed mechanism of cannabinoids in pain management.

In summary, CB1 agonists, including THC, are the key components to the analgesic effects seen with cannabis through modulation of neuronal activity in the CNS and the periphery. The effects of THC include analgesia, muscle relaxation, and antiemetic and psychotropic effects. CB2 receptor agonists, including CBD, in turn, are psycho-inactive, and may play a role in the reduction of pro-inflammatory mediators and messengers, which may contribute to the overall analgesic properties of cannabis (Figure 1).

## 6. Pharmacological and Pharmacokinetic Properties of Cannabinoids

Tetrahydrocannabinol (THC) and cannabidiol (CBD), the two most recognized and studied cannabinoids, exhibit distinct pharmacologic profiles and interactions [38]. THC metabolization is dependent on the method of consumption. When consumed orally, THC is transported to the liver, where a significant portion is either metabolized or eliminated. In the liver, enzymes CYP2C and CYP3A convert THC into other compounds, including 11-OH-THC, which is psychoactive, and 11-COOH-THC, which lacks psychoactive effects [44]. Approximately 65% or more of THC consumed orally is excreted through feces, while around 20% is eliminated via urine. Most of the consumed cannabis (80–90%) is cleared within five days, primarily as hydroxylated and carboxylated metabolites [45]. Among these, 11-COOH-THC is the primary glucuronide conjugate found in urine, whereas 11-OH-THC is predominantly excreted in feces. Residual THC and its metabolites enter the bloodstream via the heart and circulate throughout the body, with both THC and 11-OH-THC reaching the brain at the same time. Ingested THC has a bioavailability of only 4–12% and is highly lipid-soluble, accumulating in fat tissue from which it is gradually released into the bloodstream over time [46].

When cannabis is inhaled, THC and its metabolites rapidly enter the bloodstream through the lungs, with peak concentrations occurring within 6 to 10 min [47]. Inhalation provides a bioavailability ranging from 10% to 35%, depending on the user and modality. Generally, inhalation leads to more substantial psychoactive effects compared to oral consumption, as THC concentrations are higher in the brain than in the bloodstream after inhalation [48]. The plasma half-life of THC varies, lasting approximately 1 to 3 days for occasional users and extending to 5 to 13 days in chronic users [49].

The pharmacokinetics of CBD are complex, with oral bioavailability being low across different species [50]. The primary metabolites of CBD are hydroxylated 7-COOH derivatives, which are excreted either in their original form or as glucuronide conjugates [45].

The pharmacokinetics of CBD also depends on the route of administration. Inhalation yields a bioavailability ranging from 11% to 45%, with an average of 31%, while oral bioavailability in humans is approximately 6%. CBD is highly lipophilic, enabling rapid distribution to the brain, adipose tissue, and other organs [51]. Its low water solubility contributes to variability in absorption when administered in capsule form. However, CBD delivered via oil-based products or oral-mucosal methods, such as sprays or lozenges, results in more consistent pharmacokinetics. The estimated half-life of CBD ranges from 18 to 32 h [45].

## 7. Cannabinoids in Chronic Pain Management

### 7.1. THC and Synthetic Analogs

Research on THC’s effectiveness for managing chronic pain highlights its potential benefits, though its efficacy is often described as moderate and varies significantly depending on the condition. A systematic review by Whiting et al. [52] analyzed 28 studies and concluded that cannabinoids, including THC, when compared to placebo, demonstrated a modest reduction in pain (37% vs. 31%, OR 1.41) and an average reduction of pain scores on a 10-point scale by −0.41. This suggests THC can serve as a complementary treatment, especially for patients who find limited relief with conventional therapies, though the extent of pain reduction is typically mild to moderate.

### 7.2. Neuropathic Pain

For the management of neuropathic pain, results have been both promising and inconsistent. A randomized placebo-controlled trial of patients suffering from HIV-associated sensory neuropathy demonstrated more than 30% reduction in pain was reported by 52% in the inhaled cannabis group and by 24% in the placebo group [53]. Similarly, a meta-analysis by Andreae et al. [54] confirmed the efficacy of inhaled THC, showing dose-dependent pain relief in patients, reporting a reduction in pain scores in one out of every five to six patients with nerve-related pain. However, more recent research by Hansen et al. [55] found no significant impact of THC or THC/CBD delivered by oral capsule on neuropathic pain in multiple sclerosis or spinal cord injury (SCI) patients, highlighting the mixed results seen across studies. Ellis et al. [56] evaluated a cohort of patients with HIV-associated peripheral neuropathy that were treated with inhaled cannabis and showed a significant reduction in pain scores as compared to placebo, with study participants achieving a pain reduction of 30% or more when treated with cannabis vs. placebo. Further, the study authors describe a median change in pain scores (as measured with the visual analog scale) from baseline of −17 in the cannabis group vs. −4 in the placebo group. Researchers have also examined the use of cannabis for managing pain associated with diabetic neuropathy. A study conducted by Wallace et al. evaluated 16 individuals with diabetic neuropathy that were treated with inhaled cannabis to assess its short-term effectiveness and tolerability. The findings indicated a dose-dependent reduction in pain among patients who had not responded to first-line medications [57]. A study by Wilsey et al. [58] of 38 patients diagnosed with central and peripheral neuropathy (CRPS, spinal cord injury, multiple sclerosis, diabetic neuropathy) reported a dose-dependent reduction in pain with inhaled cannabis therapy.

Given the increasing amount of evidence of its efficacy, but the lack of large-scale randomized controlled trials investigating its effectiveness, The American Society of Pain and Neuroscience gives the evidence a Level 1 and Grade C recommendation for the use of cannabinoids in the treatment of neuropathic pain [59].

### 7.3. Fibromyalgia

In the treatment of fibromyalgia, THC has shown mixed outcomes. A randomized control trial by Skrabek et al. [60] demonstrated that nabilone, a synthetic THC analog delivered in an oral formulation, significantly improved pain with an average decrease in visual analog scale pain scores of −2.04 in the nabilone group vs. placebo. A randomized, double-blind, controlled study comparing nabilone to amitriptyline in the treatment of insomnia for patients with fibromyalgia demonstrated improved sleep quality in 29 patients in the nabilone group, with a reported Insomnia Severity Index improvement of 3.2 as compared to amitriptyline [61]. Conversely, Jensen et al. [62] observed inconsistent benefits, with THC providing relief for some but not all patients, pointing to variability in individual responses.

A pilot study conducted by Schley et al. evaluated the effects of medical cannabis on nine patients with fibromyalgia. The participants received orally administered dronabinol with doses ranging from 10 mg to 15 mg, which resulted in a significant reduction in reported pain levels over placebo. Those receiving THC treatment reported an average reduction in visual analog pain scores of 5.3. It should be noted that of those nine patients, five withdrew due to side effects of the therapy [63]. Additionally, Weber et al. carried out a retrospective, multicenter survey via telephone interviews of fibromyalgia patients who had been treated with an average daily dose of 7.5 mg of dronabinol over a seven-month period. They demonstrated a reduction in opioid doses and a reduction in maximum pain intensity, as rated on a numeric rating scale, from an mean of 8.7 to 4.9 in patient treated with THC [64].

The American Society of Pain and Neuroscience has reviewed the evidence regarding the use of cannabis for fibromyalgia treatment. Given multiple randomized, multicenter, placebo-controlled trials, the evidence is classified as level II. The recommendation for its use remains at grade C, as there is insufficient support from a large number of clinical trials [59].

### 7.4. Headache

In the treatment of headache disorders, cannabinoids have shown mixed results. A small randomized controlled trial [65] and multiple observational studies offered limited evidence for the efficacy of cannabis treatment of headache [66,67,68,69,70,71,72].

The level of evidence for the treatment of migraine with cannabis is poor and primarily comes from observational studies. A retrospective study by Cuttler et al. [66] of 12,293 inhaled cannabis treatment sessions for headache and 7441 for migraine demonstrated an approximate 50% reduction in headache and migraine severity. However, effectiveness appeared to decrease over time and patients appeared to use larger doses across time, suggesting tolerance to the analgesic effects of cannabis. Further, a cross-sectional study by Aviram et al. [69] of 145 patients reported that 61% of patients treated with cannabinoids had greater than a 50% reduction in their monthly migraine attack frequency with a decrease in migraine medication (opioid and triptan) consumption and disability. Another retrospective analysis by Rhyne et al. [67] of 121 adult patients with the primary diagnosis of migraine reported that abortive or preventive treatment with cannabis was associated with a reduced migraine frequency from 10.4 to 4.6 migraines per month.

The treatment of other headache disorders with cannabinoid therapy has shown mixed results. In a study by Leroux et al. [68] of 139 patients with cluster headaches, 27 (19.4%) patients reported a history of cannabis use as an attempted treatment for cluster headaches. Of those with cluster headache reporting cannabis use, 27 patients (19.4% of the total cohort) who had tried cannabis to treat cluster headache attacks, 25.9% reported some efficacy, 51.8% variable or uncertain effects, and 22.3% negative effects. Similarly, in a cohort study by Consroe et al. [73], in patients with multiple sclerosis-associated trigeminal neuralgia, more than 70% of patients reported relief with inhaled cannabis.

In a randomized, controlled trial of 26 patients with medication overuse headache, Pini et al. [65] compared nabilone (a synthetic cannabinoid) to ibuprofen. Improvements from baseline were observed with both nabilone and ibuprofen. Nabilone was significantly more effective than ibuprofen in reducing pain intensity (27.9% reduction in pain intensity) and daily analgesic intake (57.7% reduction), and nabilone was able to reduce the level of medication dependence and improve the quality of life, as compared to ibuprofen.

All other evidence for the use of cannabinoids in headache disorders comes from case reports and clinical experience, which highlights the need for additional studies on this topic [74]. Further, prospective, randomized trials are needed to determine the efficacy and safety of the use of cannabis in the treatment of headache.

### 7.5. Cancer-Related Pain

The best evidence for the use of cannabinoids in patients with cancer-related pain arises from two major randomized controlled trials by Johnson et al. [75] and Portenoy et al. [76] that demonstrated the effectiveness of nabiximols, an extract of the cannabis plant containing two cannabinoids (Δ9-tetrahydrocannabinol [27 mg/mL] and cannabidiol [25 mg/mL]) delivered as an oral spray, over placebo, for managing opioid-resistant cancer pain. Johnson et al. demonstrated a 30% reduction from baseline pain scores in patients treated with THC:CBD as compared to placebo. Conversely, a study by Lynch et al. reported no significant benefit of nabiximols compared to placebo in patients with chemotherapy-induced peripheral neuropathy. Another phase III, double-blind, randomized, placebo-controlled trial by Lichtman et al. [77] in the same population with similar intervention showed results with nabiximols not superior to placebo on the primary efficacy endpoint. Despite these mixed findings, nabiximols have been approved for cancer pain treatment in Canada and several European countries.

Opioid-based therapies are the standard approach for managing cancer-related pain; however, some patients experience pain that is unresponsive to opioids or develop significant side effects that limit their use [78]. Alternatives and adjuncts to opioids, including cannabinoids, are often considered. Given a lack of strong evidence for their use in cancer pain, guidelines do not currently recommend cannabinoids for treating cancer pain, leaving many oncologists uncertain about offering specific guidance on their use [79]. The European Society for Medical Oncology issued a grade D recommendation in its 2018 guidelines [80], citing insufficient or low-quality evidence to support cannabinoids for cancer-related pain.

Given the absence of strong data for its use in treating cancer pain, the American Society of Clinical Oncologists concluded in its updated 2024 guidelines that evidence remains insufficient to recommend for or against cannabinoids in managing cancer treatment-related toxicities or symptoms (including cancer pain), aside from the context of a clinical trial [81].

### 7.6. Multiple Sclerosis

Cannabinoids have shown some effectiveness in the treatment of multiple sclerosis (MS) symptoms [82]. The CAMS (Cannabinoids in MS) study [83] examined the effectiveness of cannabinoids for the treatment of MS symptoms in 630 patients aged 18–64 with MS-related spasticity. Patients taking Dronabinol, 2.5 mg capsules of THC, or Cannador, a cannabis extract with 2.5 mg of THC and 1.25 mg of CBD, reported significant improvements in spasticity, spasms, and sleep compared to placebo. Patients treated with cannabis extract reported a 61% reduction in spasticity and pain, and those treated with Delta9-THC reported a 60% reduction in pain and spasticity, compared to a 46% reduction in the placebo group. The MUSEC (MS and Extract of Cannabis) trial explored changes in patients’ perceived muscle stiffness in response to Cannador. The study found that patients assigned to the Cannador group for 12 weeks as opposed to the placebo group showed statistically significant improvements in spasms, pain, and sleep, with a rate of muscle stiffness relief nearly double that of placebo (29.4% vs. 15.7%; OR 2.26) [84]. Rog et al. [85] reported significant pain reduction (−2.7 vs. −2.0 on an 11-point pain scale) in multiple sclerosis (MS) patients treated with nabiximols, a THC and CBD spray as compared to placebo. A double-blinded, randomized, placebo-controlled, parallel group study looking at the effects of nabiximols on MS symptoms found that patient-rated spasticity scores were significantly reduced, and sleep quality was significantly improved in the group taking nabiximols compared to placebo [86]. Though there was also an improvement in pain in the nabiximols group, a similar improvement was seen in the placebo group.

Given the limited data, the International Association for the Study of Pain offers a grade B recommendation for the use of cannabinoids for neuropathic pain associated with multiple sclerosis [87]. The American Academy of Neurology provides the following guidelines regarding cannabis use: clinicians may consider the use of oral cannabis extract to manage spasticity symptoms and pain, excluding central neuropathic pain (Level A). THC may also be offered for similar indications (Level B). Additionally, the academy advises clinicians to inform patients that these treatments are likely ineffective for short-term objective spasticity and tremors (Level B) but may have potential benefits for long-term spasticity and pain management (Level C) [88].

### 7.7. Musculoskeletal Pain

The use of cannabis for the treatment of chronic, musculoskeletal pain remains controversial. While studies evaluating its safety and efficacy in the treatment of non-cancer, chronic pain secondary to fibromyalgia, multiple sclerosis, headache, and spinal cord injury have been performed, the data for medical cannabis use in chronic, non-cancer, musculoskeletal pain are limited [59,89]. A limited number of randomized, controlled trials investigating the efficacy and safety of cannabinoids in the treatment of pain associated with rheumatic disease demonstrated inconsistent results and non-superiority over controls [90,91]. Any conclusion regarding the efficacy of treating chronic musculoskeletal pain with cannabis is difficult to draw from the data, but the safety profile is reassuring.

## 8. Cannabinoids in Combination with Opioids

There have been several studies evaluating the potential opioid-sparing effects of cannabinoids. Multiple preclinical and animal studies have demonstrated a potential opioid-sparing effect, but a favorable translation to clinical effect has been wanted. A prior epidemiological study identified a reduction in opioid prescriptions and opioid overdose deaths in U.S. states that legalized cannabis [92]. One theory to explain this phenomenon is a potential substitution effect of cannabis for opioids or an opiate-sparing effect of cannabis.

A 2017 systematic review and meta-analysis and 2022 updated review and analysis by Nielsen et al. demonstrated an opioid-sparing effect of cannabinoids. In a review of more extensive clinical studies, there was a signal towards opioid-sparing potential, but the data were not robust, and the quality of evidence was low [93,94]. A 2021 systematic review and meta-analysis by Noori et al. [95] including eight randomized and observational studies provided very low-certainty evidence that adding cannabis reduced opioid use. The randomized trials analyzed in the study provided high-certainty evidence that cannabis addition had little or no effect on pain relief. Further, the authors concluded that opioid-sparing effects of medical cannabis for chronic pain remain uncertain due to very low-certainty evidence.

In summary, studies demonstrate, with a low quality of evidence, that cannabinoids may offer an opioid-sparing effect in patients utilizing opioids to treat pain.

## 9. Patient Perspectives and Real-World Data: Patient-Reported Outcomes

While randomized, controlled studies and their meta-analyses reduce bias and offer strong evidence, real-world data gathered from survey-based studies offer a snapshot into the real-world perceptions and effects of cannabinoids on the lives of the patients who use them as medication. Patient surveys often communicate this positive patient perception in a way that controlled studies are unable to [96]. Despite varying positions on cannabinoid efficacy in modern research, patients often perceive cannabinoids as a safer alternative to conventional analgesics [55]. A German survey conducted by the Federal Institute for Drugs and Medical Devices (BfArM) highlighted that a notable number of patients with serious illnesses viewed medical cannabis as a favorable treatment option despite the lack of robust clinical data supporting its prescription [97]. Multiple cross-sectional, survey-based studies have highlighted the ability of cannabinoids to alleviate symptoms that significantly impact quality of life in patients with a variety of medical conditions.

A 2020 systematic review by Okusanya et al. [98], including 7222 participants across nine survey-based studies, demonstrated a significant utilization of cannabinoids for opioid substitution. Patients reported a 64–75% reduction in opioid dosage when combined with opioids. One study reported a slight decrease in mean hospital admissions in the past calendar year. It decreased mean emergency department visits in the past calendar year for patients who received marijuana as an adjunct to opioids in the treatment of non-cancer chronic pain compared to those who did not receive marijuana [99].

Indeed, with any survey-based study, there is a high risk of bias due to the methods used. Even so, the survey-based data that are available signal an improvement in pain symptoms and a decrease in opioid use in patients with chronic pain who use medical cannabis.

## 10. Variability in Clinical Outcomes and Quality of the Evidence

Despite increasing research on cannabinoids for chronic pain management, the literature remains characterized by significant variability in study outcomes. This inconsistency arises from several key factors, including differences in cannabinoid formulations, delivery methods, dosages, dosing intervals, study methodologies, and patient population heterogeneity across clinical research studies. These elements contribute to challenges in interpreting findings and establishing standardized clinical recommendations.

One of the most significant challenges in cannabinoid research is the lack of standardization in formulations and delivery methods. Cannabinoids can be administered via inhalation, oral ingestion, sublingual absorption, or topical application, each of which has distinct pharmacokinetics (Table 1). For example, inhaled tetrahydrocannabinol (THC) produces rapid onset but shorter duration of effects compared to oral formulations, which are subject to first-pass metabolism and delayed systemic absorption. Variability in bioavailability and metabolism can lead to inconsistent pain relief across studies, making direct comparisons difficult. Furthermore, the ratio of THC to cannabidiol (CBD) varies widely, with some studies utilizing pure THC, others using high-CBD formulations, and some employing synthetic analogs such as nabilone or dronabinol. These differences in formulation can significantly alter analgesic efficacy and side effect profiles, complicating the interpretation of results.

Dosing inconsistency further contributes to variability in study outcomes. Cannabinoid dosages and dosing intervals are not standardized across trials, with studies employing a wide range of doses from low microgram concentrations to high milligram regimens. Additionally, the titration strategies used in clinical trials vary, with some studies allowing for individualized dose adjustments while others utilize fixed-dose regimens. Given the biphasic nature of cannabinoid effects, where lower doses may be therapeutic while higher doses can lead to diminishing returns or adverse effects, the lack of a uniform dosing protocol presents a major confounding variable.

Beyond pharmacologic considerations, pain itself is inherently subjective and difficult to quantify, further complicating the assessment of cannabinoid efficacy. Pain perception is influenced by multiple factors, including psychological state, baseline pain sensitivity, and comorbid conditions such as anxiety or depression. The use of different pain assessment scales, ranging from numerical rating scales (NRS) to visual analog scales (VAS) and qualitative patient-reported outcomes, introduces additional variability. Furthermore, placebo responses in pain studies are notoriously high, often exceeding 30% in some trials, which may obscure true treatment effects and lead to conflicting conclusions.

Small sample sizes and study population heterogeneity also contribute to inconsistent findings. Many cannabinoid studies involve relatively small cohorts, limiting statistical power and the generalizability of results. Patient populations in these trials often include individuals with diverse pain etiologies, ranging from neuropathic pain and fibromyalgia to cancer-related and musculoskeletal pain. The underlying mechanisms of these conditions differ significantly, and cannabinoids may exert varying degrees of efficacy depending on the pain subtype. Additionally, genetic and metabolic differences in cannabinoid receptor function and endocannabinoid system activity among individuals may result in variable responses to treatment.

Finally, methodological inconsistencies across studies further hinder direct comparisons. Variations in study design, including crossover versus parallel-group trials, duration of treatment, and control conditions (e.g., placebo vs. active comparator), contribute to discrepancies in reported outcomes. Some studies incorporate adjunctive therapies such as opioids or nonsteroidal anti-inflammatory drugs (NSAIDs), while others evaluate cannabinoids as monotherapy, leading to differences in observed analgesic effects.

Addressing these challenges requires greater standardization in future research, including uniform cannabinoid formulations, clearly defined dosing regimens, standardized pain assessment tools, and larger, well-powered randomized controlled trials (RCTs) with long-term follow-up. By mitigating these sources of variability, future studies can generate more reliable data to guide clinical decision-making regarding cannabinoid-based pain management.

## 11. Potential Risks of Cannabinoid Use

Cannabinoids are not devoid of risks. Their use carries a potential for addiction, psychiatric effects, and drug–drug interactions.

THC and synthetic cannabinoids are moderately addictive [100]. These compounds activate CB1 receptors to increase dopamine in the brain’s reward circuit, which can lead to craving, drug-seeking behavior, and dependence [101]. With expanding marijuana legalization across the U.S., maladaptive use patterns are of increasing concern.

A 2023 cross-sectional study in JAMA found that cannabis use disorder affected up to 21% of users in Washington state, where recreational marijuana is legal [102]. Of note, those using cannabis for nonmedical reasons had a higher risk of moderate–severe cannabis use disorder. Marijuana use at younger ages and daily consumption are additional risk factors for addiction [103].

CBD, by contrast, has a very low risk of abuse given its minimal psychiatric effects [104]. Preclinical studies suggest CBD may reduce drug-seeking behavior in the context of alcohol, opioids, and methamphetamine. A 2022 systematic review also found that CBD might alleviate opioid cravings, cigarette use, and cannabis withdrawal, highlighting its therapeutic potential in humans [105].

Acute and chronic psychiatric effects are another risk to consider. A 2020 meta-analysis of 15 studies found that THC significantly increased the severity of positive psychotic symptoms (e.g., hallucinations, disorganized thinking), negative symptoms (e.g., emotional withdrawal), and general symptoms (e.g., anxiety, depression) in healthy participants [106]. While these acute effects are dose-dependent and transitory [107], they are essential to consider in patients with preexisting mental health conditions.

Chronic cannabinoid use has been linked to an elevated risk of mood disorders, schizophrenia, and cognitive impairment. A 2016 meta-analysis of 66,816 individuals found an association between increasing total cannabis consumption and risk of psychosis or schizophrenia [108]. Another systematic review showed evidence that chronic cannabis use might impair memory, attention, and concentration even after 14 days of abstinence [109]. Whether recovery might come with more extended periods of sobriety remains uncertain.

Although cannabinoids have been studied for therapeutic benefit in depression, anxiety, ADHD, and PTSD, the evidence to support this is scarce and low-quality [110]. One small study (n = 6) suggested CBD may attenuate THC-induced psychotic symptoms, but these findings were unable to be replicated by three more extensive studies [106].

The COMPASS trial (Cannabis for Management of Pain: Assessment of Safety Study) examined the safety profile of cannabis in 215 Canadian patients with chronic pain. The study found that individuals in the group using standardized cannabis (12.5% THC) experienced significantly more non-serious adverse events compared to the non-user group [111]. These non-serious adverse events included neurologic, psychiatric, or respiratory symptoms. More common, non-serious adverse side effects included headache, nasopharyngitis, nausea, somnolence, dizziness, upper respiratory tract infections, vomiting, and cough while less common adverse events included amnesia, euphoric mood, sweating, and paranoia. While cannabis was linked to these non-serious effects, the study found no significant association between its use and serious adverse events.

Because CYP3A4, CYP2C9, and CYP2C19 enzymes metabolize cannabis, there is a possibility for interactions with similarly processed medications [112]. A 2022 systematic review found clinically significant risks when combining cannabis with warfarin (categorized as very high risk), buprenorphine (high risk), and tacrolimus (high risk) [113]. Two 2024 systematic reviews further investigated cannabis–drug interactions. The first review of 54 articles found antiepileptics, clobazam, warfarin, and tacrolimus as the most likely to interact with cannabis [114]. A second review of 603 cannabis users noted adverse reactions such as increased bleeding risk, altered mental status, difficulty inducing anesthesia, and gastrointestinal issues by combining cannabis with warfarin, valproate, tacrolimus, and sirolimus [112]. These studies underscore the importance of carefully monitoring patients for potential adverse effects when cannabis is used alongside other high-risk medications.

Beyond these side effects, cannabis can lead to other challenges, including the development of tolerance and physical dependence with continued use. Though the rate of physical dependence on cannabis (9%) is lower than that of tobacco (67.5%), alcohol (22.7%), and cocaine (20.9%), individuals using cannabinoids remain at risk for developing cannabis withdrawal syndrome [115]. Frequent users of cannabis often develop physical dependence with chronic use and experience withdrawal symptoms including irritability, insomnia, restlessness, hot flashes, and cramping.

Other studies have reported myriad side effects affecting nearly every organ system (Table 2). These side effects include, but are not limited to, cognitive deficits, anxiety, psychosis [106,108,116], reproductive system effects [117,118], alterations in fetal development [117], airway irritation and chronic bronchitis, increased risk of lung cancer [119], risk of insulin resistance and metabolism changes [120], gastrointestinal effects [121], and cardiovascular risks [122] as possible side effects of cannabinoid use. Case reports of chronic cannabis users have reported adverse cardiovascular risks for myocardial infarctions, tachycardia, dysrhythmias, hypotension, and orthostatic hypertension [123]. There is also growing evidence that chronic and acute cannabis intoxication is associated with strokes and cardiovascular events [122,124]. Additionally, meta-analyses have identified a dose–response relationship between cannabis use and the risk of psychotic disorders [125].

## 12. Guidance for Physicians on the Safety of Cannabinoids Compared to Other Analgesics

When considering cannabinoids for chronic pain management, physicians should weigh not only their effectiveness but also their safety profile against other commonly used analgesics, including opioids, NSAIDs, and gabapentinoids. While cannabinoids may offer therapeutic benefits, their unique risks and side effects necessitate careful patient selection and monitoring.

Opioids: Given the well-documented risks of opioid use, including respiratory depression, dependence, cognitive effects, gastrointestinal disturbance, and overdose mortality [126], cannabinoids may serve as an adjunct or alternative, particularly in patients requiring long-term pain management. Unlike opioids, cannabinoids do not cause fatal respiratory depression, but chronic use may lead to cannabis use disorder, cognitive impairment, and psychiatric effects in predisposed individuals. Physicians should consider cannabinoids in opioid-tolerant patients seeking alternative therapies with a lower overdose risk.

Nonsteroidal anti-inflammatories (NSAIDs): NSAIDs such as ibuprofen and naproxen are effective for inflammatory pain but carry risks of gastrointestinal bleeding, platelet inhibition, renal impairment, and cardiovascular complications, especially in older adults or those with preexisting conditions [127]. Cannabinoids, while not associated with these specific risks, can impair cognition and cause dizziness, which may affect patient safety. Notably, cannabis has been associated with increased risk of cardiac events and stroke [122]. Prescribers should evaluate whether cannabinoids are suitable for patients who are at high risk for NSAID-related complications.

Gabapentinoids (Gabapentin and Pregabalin): Gabapentinoids are widely used for neuropathic pain but have been linked to sedation, sleep disturbances, dizziness, and an increasing potential for misuse and dependence [128]. Cannabinoids may offer comparable neuropathic pain relief while avoiding the respiratory depression risk seen with high-dose gabapentinoid use. However, prescribers should carefully monitor patients for psychoactive effects and ensure proper titration.

Physicians should take a patient-centered approach when considering cannabinoids, assessing individual pain conditions, comorbidities, and potential drug interactions. Shared decision-making, patient education, and gradual dose adjustments are crucial to optimizing safety and efficacy. Given the variability in cannabinoid formulations and dosing, prescribers should remain informed on emerging clinical evidence and evolving regulatory guidelines to integrate cannabinoids responsibly into pain management strategies.

In conclusion, cannabinoids carry a moderate addiction potential, short- and long-term psychiatric risks, the possibility for serious drug interactions, and a potential increased risk of stroke and cardiovascular events. It is important to carefully consider these risks against expected benefits with patients while treating their pain.

Psychoactivity of Cannabinoids and Strategies to Preserve Analgesia While Minimizing Adverse Effects

One of the primary concerns regarding the use of cannabinoids in chronic pain management is their psychoactive effects, primarily mediated by tetrahydrocannabinol (THC) through CB1 receptor activation in the central nervous system. These effects can include euphoria, cognitive impairment, sedation, dizziness, anxiety, and, in some cases, paranoia or psychosis, particularly at higher doses [116]. While these psychoactive properties may be undesirable for many patients, the challenge in cannabinoid-based pain management lies in preserving the analgesic benefits while minimizing central nervous system side effects.

Several approaches have been explored to achieve this balance:

CBD, unlike THC, does not directly activate CB1 receptors and lacks significant psychoactive effects. Preclinical and clinical studies suggest that CBD may have analgesic and anti-inflammatory properties, possibly through modulation of CB2 receptors, inhibition of pro-inflammatory cytokines, and interaction with serotonin receptors [40,42]. Some research indicates that CBD may mitigate THC-induced psychoactive effects by modulating CB1 receptor activity [129]. However, while CBD shows promise for pain relief, its efficacy as a sole analgesic remains less established compared to THC.

## 13. THC:CBD Ratios and Synergistic Effects

Studies suggest that formulations containing both THC and CBD in specific ratios (e.g., 1:1 or 1:4) may provide effective analgesia while reducing THC-related psychoactivity [130]. Nabiximols (Sativex), an oromucosal spray containing a balanced THC:CBD ratio, has been studied extensively for conditions such as multiple sclerosis-related spasticity and neuropathic pain, demonstrating efficacy with a more tolerable psychoactive profile [83,84,85,86].

## 14. Alternative Cannabinoids with Reduced Psychoactivity

Other phytocannabinoids, such as cannabigerol (CBG) and tetrahydrocannabivarin (THCV), are being investigated for their potential analgesic effects without strong psychoactive properties [131]. THCV, in particular, acts as a CB1 receptor antagonist at lower doses and may counteract some of THC’s psychoactive effects [38]. These cannabinoids represent a potential avenue for pain relief with fewer central nervous system side effects.

## 15. Route of Administration and Dosing Strategies

The delivery method of cannabinoids can significantly impact psychoactivity. Inhalation and oral administration result in higher systemic THC concentrations and increased central effects [46,47,48,49]. In contrast, transdermal or topical formulations may allow localized pain relief with limited systemic absorption, reducing psychoactive effects [132].

While the psychoactivity of cannabinoids remains a challenge in clinical pain management, strategies such as optimizing THC:CBD ratios, targeting peripheral cannabinoid receptors, exploring alternative cannabinoids, and utilizing controlled-release or localized delivery methods offer promising avenues for minimizing adverse effects while preserving analgesic efficacy. Future research should focus on refining these approaches to enhance the safety and acceptability of cannabinoid-based pain therapies.

## 16. Conclusions

Cannabinoids offer a promising yet complex alternative for chronic pain management, particularly given the limitations and risks of traditional therapies like opioids. Evidence suggests moderate efficacy in conditions including neuropathic pain, fibromyalgia, cancer-related pain, and multiple sclerosis-related spasticity, with potential opioid-sparing effects when conventional options fall short. Patient-reported outcomes often reflect a favorable perception of cannabinoids as safer alternatives, though robust clinical validation remains limited. However, research gaps persist due to small sample sizes, short study durations, and methodological inconsistencies, highlighting the need for large-scale, randomized controlled trials with long-term follow-up to confirm efficacy and safety across diverse populations. Regulatory barriers, particularly in the U.S. where cannabis remains a Schedule I substance, further hinder clinical research and patient access, though evolving policies offer hope for progress. Moving forward, a stronger evidence base will be essential for shaping clinical guidelines, refining prescribing practices, and optimizing dosing strategies to maximize benefits while minimizing risks. The future for cannabis research is bright, and as regulatory frameworks adapt to balance access and oversight, cannabinoids may transition from an experimental adjunct to a well-established option in chronic pain care, provided scientific rigor and evidence-based policymaking remain at the forefront.

## Figures and Tables

**Table 1:** Available cannabis administration products.

Administration Method	Description	Available Products
Oral Administration		
Oral Solutions	Cannabis extracts in liquid form, usually administered via dropper	Dronabinol oral solution (Syndros™, Benuvia Therapeutics, Round Rock, TX, USA)
Mucosal Sprays	Liquid formulations for spray application under the tongue or in the mouth	Nabiximols (Sativex™, GW Pharmaceuticals, Cambridge, UK)
Capsules	Orally administered capsules containing synthetic or natural cannabis compounds	Dronabinol capsule (Marinol™, Solvay Pharmaceuticals, Neder-Over-Heembeek, Brussels, Belgium) Nabilone (Cesamet™, Bausch Health Companies, Laval, QC, Canada) Cannabics™ (Cannabics Pharmaceuticals, Bethesda, MD, USA)
Tablets	Solid form containing synthetic cannabis	Namisol™ (Echo Pharmaceuticals, Leiden, The Netherlands)
“Edibles”	Food or drinks infused or paired with cannabis oils, extracts or flower	Gummies, candies, baked goods, tinctures, and beverages
Inhaled Administration		
Vaporized	Heating cannabis in a vaporizer for inhalation	Vaporizer “pens”, portable vaporizers
Smoked	Inhalation of cannabis leaves, resin, or hashish	Cigarette, pipe, hookah, bong
Other Routes		
Topicals	Cannabis products infused into topical delivery systems	Cannabis oil-infused creams, patches, and topical oils
Intramuscular Injections	Synthetic cannabis compounds administered via injection	Levonantradol

**Table 2:** Risks and side effects of cannabis use.

Body System	Potential Risks and Side Effects	Notes	References
Central Nervous System (CNS)	Cognitive impairment, dizziness, sedation, impaired memory, attention deficits, psychosis	Higher THC concentrations increase psychoactive effects	[52,83,106,108,111,115,116]
Psychiatric	Anxiety, paranoia, depression, exacerbation of schizophrenia, cannabis use disorder	Risk likely varies based on genetic predisposition and on frequency of use	[103,104,106,107,109]
Cardiovascular	Tachycardia, orthostatic hypotension, increased risk of myocardial infarction, stroke (in heavy users)	Effects may be more pronounced in older adults or those with existing cardiovascular disease	[122,123,124]
Respiratory	Chronic bronchitis, cough, airway inflammation, potential for lung injury, increased risk of lung cancer (if smoked)	Vaporized or oral formulations may reduce respiratory risks	[119]
Gastrointestinal	Nausea, vomiting, cannabinoid hyperemesis syndrome, adult intussusception	More common with chronic, high-dose use	[111,121]
Endocrine and Metabolic	Altered glucose metabolism, potential impact on insulin sensitivity, appetite stimulation	Evidence suggests differential effects of THC vs. CBD on metabolism	[120]
Reproductive System	Decreased sperm count and motility, potential impact on ovulation and fetal development	Effects on fertility are still under investigation	[117,118]
Immune System	Immunomodulatory effects, potential suppression of immune response	Clinical significance remains uncertain	[39,41]
Fetal Development	Fetal growth restriction, low birth weight, small for gestational age, increased NICU admission, increased incidence of neurocognitive and neuropsychiatric disorders	Demonstrated in fetuses of mothers that use cannabis during pregnancy	[117]
